# A Medico-Legal Dilemma in Emergency Airway Management in a Rapidly Progressive Parapharyngeal Space Abscess: A Case Report

**DOI:** 10.7759/cureus.109109

**Published:** 2026-05-18

**Authors:** Paromita Roy Chandra

**Affiliations:** 1 Otolaryngology - Head and Neck Surgery, Diamond Harbour Government Medical College, Kolkata, IND

**Keywords:** airway compromise, deep neck space infection, emergency decision-making, medico-legal dilemma, parapharyngeal abscess, tracheostomy

## Abstract

Deep neck space infections are potentially life-threatening due to their rapid progression and risk of airway compromise. Emergency airway management in such cases may be further complicated by consent-related challenges.

A 28-year-old previously healthy woman presented with dysphagia following recent hemodialysis with right-sided central venous catheterization. Her symptoms progressed to neck swelling, hoarseness, and respiratory distress. Fiber-optic examination suggested deep neck space involvement, and contrast-enhanced CT confirmed a right parapharyngeal space abscess. Despite initiation of intravenous antibiotics and planned drainage, the patient deteriorated rapidly with signs of impending airway compromise.

Emergency tracheostomy and surgical drainage were planned. However, consent for tracheostomy was refused by the patient’s relatives despite detailed counselling. Following urgent multidisciplinary deliberation and in view of life-threatening airway obstruction, a decision was made to proceed with emergency airway management.

Tracheostomy and drainage were successfully performed, resulting in stabilization and subsequent clinical recovery.

This case highlights the importance of early recognition of airway compromise in deep neck infections, the need for timely and decisive airway management, and the ethical challenges encountered when life-saving interventions are refused.

## Introduction

Deep neck space infections (DNSIs) are serious and potentially life-threatening infections involving the fascial planes and deep cervical compartments of the neck. Although the widespread use of antibiotics has reduced their incidence, DNSIs continue to be associated with considerable morbidity and mortality due to their rapid progression and potential for airway compromise, septicemia, and mediastinal extension [1,2]. Early diagnosis and prompt management remain essential to prevent fatal complications.

The deep cervical fascia divides the neck into several potential spaces, including the parapharyngeal, retropharyngeal, and submandibular spaces. Among these, the parapharyngeal space is of particular clinical significance because of its close anatomical relationship to major neurovascular structures such as the carotid artery, internal jugular vein, cranial nerves IX-XII, and the upper airway [3]. Infection within this space can spread rapidly to adjacent compartments and may result in airway obstruction, descending necrotizing mediastinitis, vascular complications, and septic shock [4,5].

The etiology of DNSIs has evolved over time. Traditionally, odontogenic and tonsillopharyngeal infections were considered the most common causes [6]. However, with the increasing use of invasive medical procedures, iatrogenic causes such as endotracheal intubation, dental procedures, and central venous catheterization are being recognized more frequently [7]. Central venous catheter-related infections may act as a nidus for bacterial spread into the deep cervical spaces, particularly in critically ill or immunocompromised patients.

Clinical presentation may initially be subtle and nonspecific, with symptoms such as fever, neck pain, dysphagia, odynophagia, muffled voice, or neck swelling. However, rapid deterioration may occur due to edema and abscess formation, resulting in respiratory distress and impending airway obstruction [8]. Because of this unpredictable clinical course, close monitoring and early recognition of airway compromise are crucial.

Contrast-enhanced computed tomography (CT) is considered the imaging modality of choice for evaluating DNSIs, as it accurately delineates the extent of infection, identifies abscess formation, and aids in surgical planning [9]. Management typically includes broad-spectrum intravenous antibiotics, drainage of purulent collections, and timely airway stabilization when indicated. Airway management remains the cornerstone of treatment, and delays in securing the airway significantly increase mortality risk [10].

In addition to the clinical challenges, emergency airway intervention may occasionally present ethical and medico-legal dilemmas, particularly when consent for life-saving procedures is refused by relatives or caregivers. Physicians are then required to balance patient autonomy, emergency medical ethics, and legal responsibility while ensuring the patient’s best interests are protected. This case highlights both the clinical urgency and medico-legal complexities involved in managing a rapidly progressive parapharyngeal abscess following central venous catheterization.

## Case presentation

A 28-year-old previously healthy woman presented with dysphagia of acute onset. She had a recent history of acute renal illness requiring hemodialysis, during which a right-sided central venous catheter had been placed. The patient reported that her symptoms began shortly after a dialysis session.

On initial evaluation, fiber-optic laryngoscopy revealed medialization of the right anterior tonsillar pillar and tonsil, raising suspicion of a deep neck space infection. A contrast-enhanced CT scan of the neck was advised.

Within 24 hours, the patient developed progressive right-sided neck swelling, worsening dysphagia, and pain. The central venous catheter was removed due to suspicion of a catheter-related complication.

**Figure 1:**
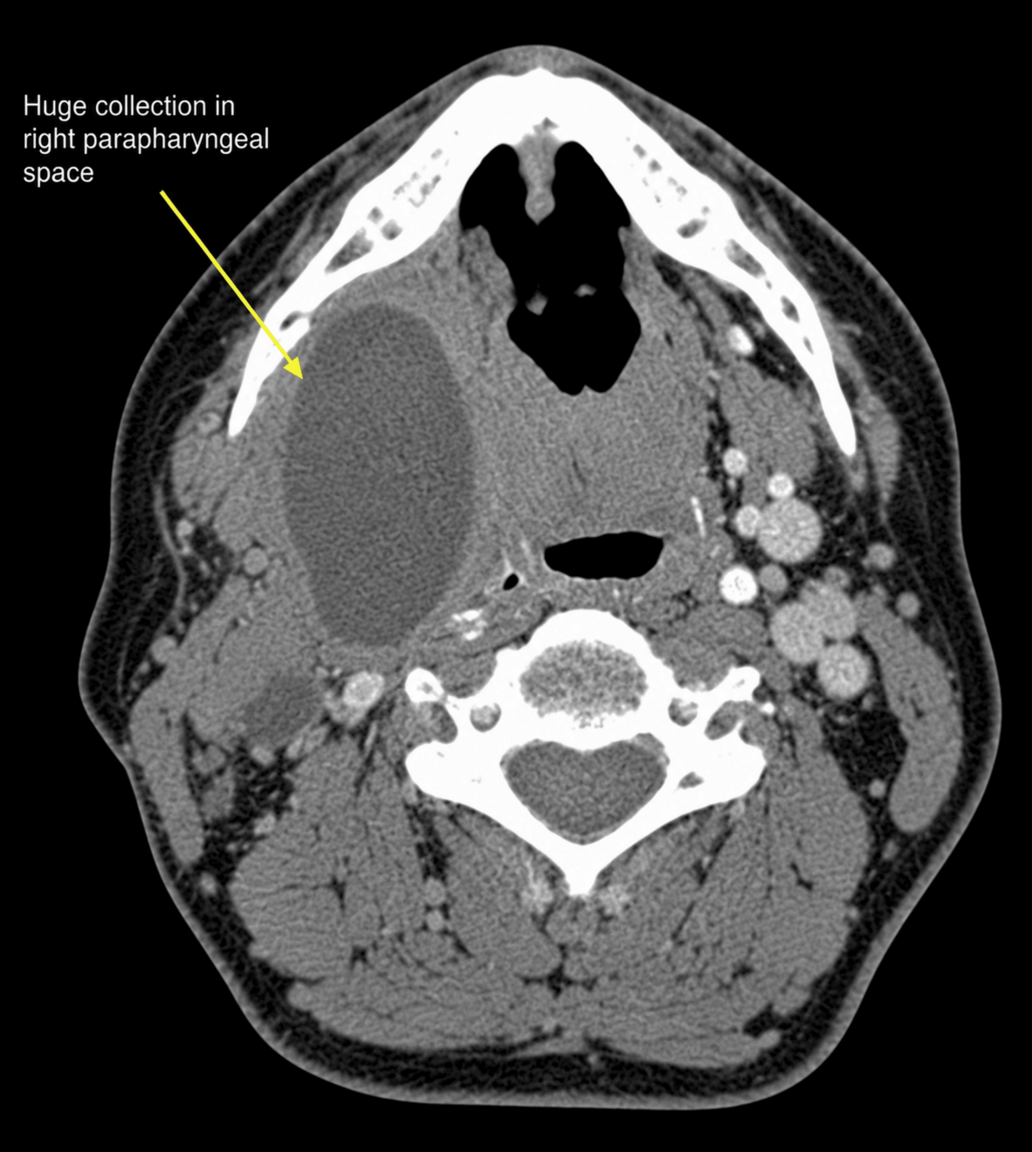
Contrast-enhanced CT scan of the neck showing parapharyngeal space abscess

**Figure 2:**
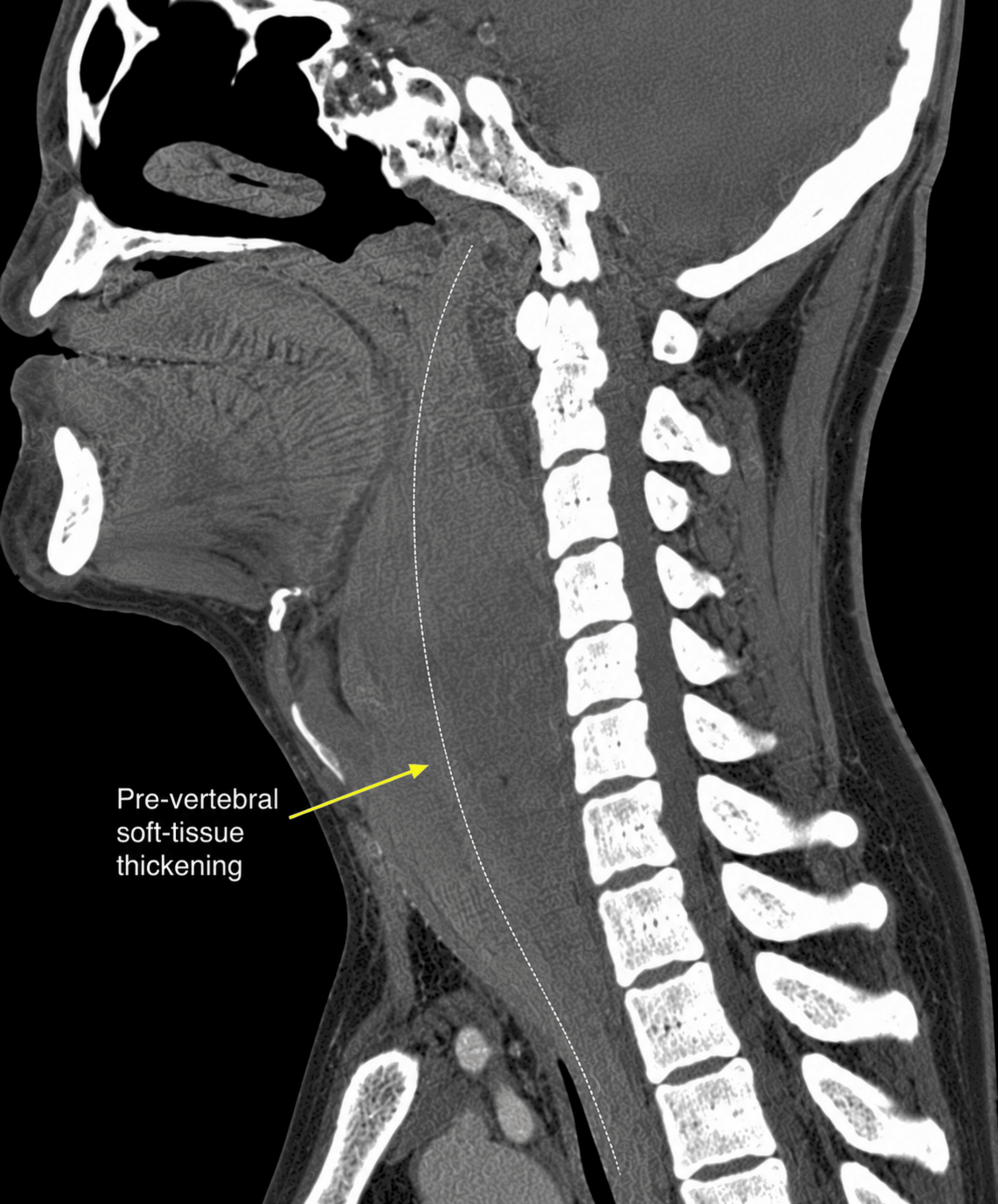
Pre-vertebral soft tissue thickening on sagittal view of the CT scan of neck

CT imaging revealed a collection in the right parapharyngeal space extending into the peritonsillar region, consistent with a deep neck space abscess (Figures 1, 2). Intravenous antibiotics and analgesics were initiated, and drainage was planned.

Prior to the scheduled intervention, the patient deteriorated rapidly, developing hoarseness of voice, increasing neck swelling, tachycardia, and severe respiratory distress, necessitating transfer to the intensive care unit. Clinical findings suggested impending airway compromise.

Emergency tracheostomy followed by incision and drainage was planned. However, consent for tracheostomy was refused by the patient’s relatives despite detailed counselling regarding the risk of airway obstruction and death. The relatives requested transfer to another facility, which was considered unsafe due to the patient’s critical condition.

An urgent multidisciplinary discussion was conducted. In view of the life-threatening situation and high risk of complete airway obstruction, a decision was made to proceed with emergency airway intervention.

Emergency tracheostomy and surgical drainage were performed successfully, resulting in stabilization and subsequent recovery.

## Discussion

Deep neck space infections are associated with significant morbidity and mortality, primarily due to airway compromise and spread to adjacent vital structures [4,5]. The parapharyngeal space, due to its anatomical continuity with other deep neck compartments, allows rapid extension of infection and early airway involvement [6].

In this case, the patient demonstrated rapid clinical deterioration from initially mild symptoms such as dysphagia to severe respiratory distress. Warning signs - including progressive neck swelling, voice changes, and respiratory difficulty - indicated impending airway obstruction.

Although a temporal association with central venous catheterization was noted, a definitive causal relationship cannot be established in the absence of microbiological or procedural confirmation.

Airway management remains the most critical component in the treatment of DNSIs. Delayed airway intervention is associated with significantly increased morbidity and mortality [7,8].

Emergency clinicians should maintain a high index of suspicion in head and neck infections, as seemingly mild symptoms may rapidly progress to life-threatening airway compromise [9-11].

This case also highlights the complexity of decision-making when consent for life-saving procedures is refused. In emergency situations where there is an immediate threat to life, clinicians may proceed under the emergency exception to informed consent, guided by ethical frameworks and institutional policies rather than broad legal assumptions [12].

## Conclusions

This case highlights the rapidly progressive and potentially life-threatening nature of deep neck space infections, particularly parapharyngeal abscesses, and underscores the importance of early recognition and timely airway management. It emphasizes that clinical deterioration may occur despite initially mild symptoms, necessitating vigilant monitoring and proactive airway planning. While a possible iatrogenic association was observed, this should be interpreted cautiously in the absence of definitive evidence.

The case also illustrates the ethical challenges encountered when life-saving interventions are refused. In such situations, prompt, well-documented, and multidisciplinary decision-making - guided by established ethical principles - is essential. Ensuring airway security and patient survival remains the foremost priority in managing such high-risk clinical scenarios.
